# The Impact of Maternal Hypothyroidism during Pregnancy on Minipuberty in Boys

**DOI:** 10.3390/jcm12247649

**Published:** 2023-12-13

**Authors:** Karolina Kowalcze, Robert Krysiak, Anna Obuchowicz

**Affiliations:** 1 Department of Pediatrics in Bytom, Faculty of Health Sciences in Katowice, Medical University of Silesia, Stefana Batorego 15, 41-902 Bytom, Poland; aobuchowicz@sum.edu.pl; 2 Department of Internal Medicine and Clinical Pharmacology, Medical University of Silesia, Medyków 18, 40-752 Katowice, Poland

**Keywords:** genital organs, hypothalamic–pituitary–gonadal axis, hypothyroidism, infants, pregnancy complications, saliva

## Abstract

Minipuberty is a period of increased reproductive axis activity in infancy, which seems to be implicated in the postnatal development of male genital organs. Impaired thyroid function during pregnancy is associated with an increased risk of prenatal, perinatal, and postnatal complications. The aim of this study was to investigate whether the presence of hypothyroidism during pregnancy modulates the course of male minipuberty. We compared three matched groups of male infants: sons of women with hypothyroidism uncontrolled or poorly controlled during pregnancy (group A), male offspring of women treated over the entire pregnancy with adequate doses of levothyroxine (group B), and sons born to women with no evidence of thyroid disease (group C). Salivary levels of testosterone, androstenedione, dehydroepiandrosterone sulfate, estradiol, progesterone, and 17-hydroxyprogesterone, as well as urine concentrations of FSH and LH, were assessed once a month in the first 6 months of life, and once every two months between months 6 and 12. Gonadotropin and testosterone levels during the first 6 months of life were lower in group A than in groups B and C. Differences in testosterone and gonadotropin levels were accompanied by similar differences in penile length and testicular volume. Concentrations of the remaining hormones did not differ between the study groups. The obtained results suggest that untreated or undertreated maternal thyroid hypofunction in pregnancy has an inhibitory effect on postnatal activation of the hypothalamic-pituitary-testicular axis and genital organ development in their male offspring.

## 1. Introduction

Minipuberty is a term describing transient activation of the hypothalamic–pituitary–gonadal axis occurring in both sexes during the first few months of life. In healthy boys, follicle-stimulating hormone (FSH), luteinizing hormone (LH), and testosterone levels are highest between 4 and 12 weeks of life and decline thereafter, reaching prepubertal values between 4 and 6 months of life (testosterone) or between 6 and 9 months of life (gonadotropins) [1,2,3]. The biological significance of minipuberty has not been fully understood. Despite enhanced production, the action of testosterone is limited by a simultaneous increase in sex hormone-binding globulin [4] and by the lack of the androgen receptor in some types of cells (particularly in Sertoli cells) [5]. However, minipuberty is assumed to play an important role in the postnatal development of genital organs in males, explaining accelerated penile growth [6] and testicular enlargement [7] in early childhood (the first 3–5 months of life). Transient activation of the gonadal axis may also be implicated in the regulation of growth velocity [8], may determine body weight and adipose tissue distribution [9], and may play a role in the development of language and emotional competencies [10].

To the best of our knowledge, no human study has assessed the association between maternal thyroid disorders during pregnancy and hypothalamic–pituitary–gonadal axis activation in early childhood. However, some indirect pieces of evidence suggest that impaired functioning of the thyroid gland may have an impact on the reproductive axis in the developmental age. Boys born to mothers with hyperthyroidism were characterized by accelerated pubertal development, which was not observed in girls and children born to mothers with thyroid hypofunction [11]. Thyroid hypofunction diagnosed in the prepubertal age delayed puberty and resulted in incomplete isosexual precocity (development of breast and internal genitalia in girls and increased testicular volume in boys without adrenarche) [12]. In turn, experimentally-induced thyroid hypofunction in pregnant rodents caused quantitative changes in testicular histology in their male offspring [13,14]. Lastly, untreated or inadequately treated hypothyroidism is a well-evidenced risk factor for preterm delivery [15], while the latter was found to affect the chronology of minipuberty (premature male infants were characterized by higher LH and testosterone concentrations and by faster growth of the penis and testes than full-term infants) [16]. The lack of targeted studies and a likely important physiological role of the postnatal gonadal axis activation encouraged us to investigate whether the presence of hypothyroidism during pregnancy and its compensation affect the course of male minipuberty.

## 2. Materials and Methods

This single-center, prospective, matched cohort study was conducted between April 2022 and September 2023. The research protocol was evaluated and approved by the Bioethical Committee of the Medical University of Silesia, and the study was guided by the ethical principles of the 1964 Declaration of Helsinki. Written informed consent was obtained from the parents on behalf of the child after they had been fully informed by the investigator of the nature and potential risks. Because of its observational nature, clinical trial registration was not applicable.

### 2.1. Participants

The participants were recruited from among apparently healthy boys born in the past month. All potential participants were sent to our outpatient department by local healthcare providers (neonatologists and pediatricians) cooperating with the investigators and were sons of women consulted during pregnancy by an endocrinologist. All infants and their mothers were white Polish Caucasians inhabiting the Upper Silesia, the urban area situated in the southern part of Poland.

The children were allocated by the principal investigator (K.K.), who specializes in pediatric medicine and endocrinology, into one of three groups. An a priori sample size calculation showed that we needed 25 infants in each group to detect a 25% difference in salivary testosterone concentrations versus the control group (the primary endpoint) with 80% power and α error of 0.05. Group A included sons of women with hypothyroidism, untreated or undertreated during pregnancy. Group B included male offspring of women who, over the entire pregnancy, were treated with adequate doses of levothyroxine (50–150 μg daily) due to previously diagnosed hypothyroidism. Lastly, group C (the reference group) enrolled sons of healthy women with no evidence of thyroid disease. Infants were assigned to group A if maternal plasma TSH levels on two or more measurements during pregnancy (at least six weeks apart) were above 4.0 mU/L. In pregnant women meeting these criteria, according to American Thyroid Association guidelines [17], levothyroxine treatment is either obligatory (thyroid peroxidase-positive women) or should be considered (thyroid peroxidase-negative women), and TSH levels above this threshold value are higher than target maternal TSH concentrations. Hypothyroidism was considered adequately treated if all TSH measurements during pregnancy (not less than three) were within the reference range (values between the lower reference limit reduced by 0.4 mU/L and 4.0 mU/L in the first trimester; 0.5–4.0 mU/L in the second and third trimester). Mothers were considered healthy if (1) TSH levels on two or more measurements during the last 12 months (including at least one measurement in the first trimester of pregnancy) were between the lower reference range reduced in the first trimester of pregnancy by 0.4 mU/L and 4.0 mI/L; (2) free thyroid hormone levels and thyroid antibody titers (if assessed) were within the reference range; (3) they did not experience hypothyroid symptoms before or during the pregnancy; and (4) during the pregnancy (if assessed) and at the time of recruitment their thyroid gland was of normal size and echogenicity. Sons of women (1) with only one abnormal TSH result; (2) with isolated hypothyroxinemia (defined as free thyroxine levels below 10.1 pmol/L and normal TSH levels), or (3) with discordant laboratory findings during pregnancy were not considered eligible for the study. To exclude congenital hypothyroidism in the study population, only infants with TSH levels below 12 mIU/L in dried whole blood spots (obtained on day 3–5 of life) and without sonographic features of thyroid dysgenesis (aplasia, hypoplasia, or ectopia) were considered eligible for enrollment. To obtain three study groups matched for maternal age, education, occupational activity, gestational age of delivery, and the number of deliveries, the participants were selected among a larger population of apparently healthy infant boys. The algorithm matching patients was based on the minimum Euclidean distance rule. All information on maternal age and gestational age of delivery (as well as on the number of deliveries, birth order, smoking, mean body mass index during pregnancy, mean blood pressure during pregnancy, and mean TSH concentrations) was extracted from mothers’ and infants’ medical records stored online in a digital format. The necessary information about education and occupational activity was obtained by interviewing the mothers during the first visit.

Patients were excluded if they had major congenital anomalies, genetic syndromes, metabolic disorders, congenital infections, or cryptorchidism; had developed birth asphyxia; were in utero exposed to alcohol; were born before week 36 of pregnancy; received any chronic pharmacotherapy; were offspring of parents with genetic syndromes; had mothers with other chronic disorders diagnosed before pregnancy, during pregnancy or postpartum; were offspring of women hospitalized during pregnancy because of acute complications; or had mothers receiving during pregnancy any chronic pharmacotherapy (except for levothyroxine and micronutrient and vitamin preparations for pregnant women).

### 2.2. Study Design

Follow-up visits took place once a month in the first 6 months of life and once every two months between months 6 and 12. At each visit, the investigator asked the parents about progress in reaching age-appropriate developmental milestones, the child’s condition, and any medical treatment (including vaccinations), performed a detailed physical examination, and interpreted the results of laboratory tests (if performed). Anthropometric measurements were recorded, and the biological material for laboratory tests was collected only if the child was considered healthy and did not receive any drug, except for obligatory vaccines, during the last 10 days. The final analysis included only patients assessed in at least 6 time points.

Anthropometric evaluation was based on standard measurements. Recumbent length was measured from the crown of the head to the foot’s heel to the nearest 0.1 cm using a portable infantometer (Seca, Hamburg, Germany). Body weight was measured on a digital scale with ±10 g precision (Seca 834, Hamburg, Germany). Body mass index was calculated as weight in kilograms divided by squared length in meters. In turn, head circumference was measured by passing the tape around the head, placing it anteriorly on the forehead just above the eyebrows and posteriorly at the most posterior protuberance of the back of the head.

The penile length was measured using a calibrated tape from the pubopenile skin junction to the top of the penis, excluding the foreskin. Before each measurement, the penis was fully stretched to the point of increased resistance. The final result was averaged from three independent measurements.

All patients were also examined by a standard testicular sonographic technique using a 5-MHz to 12-MHz transducer (Esaote MyLab Six, Genoa, Italy). Separate transverse and longitudinal images of each testis were obtained, and the largest diameters were used to calculate the volume. The epididymis was not included in the volume measurement. The volume of each testis was calculated using Lambert’s equation (length × width × height × 0.71), which provides better accuracy for testicular volume assessment than other sonographic formulas [18,19]. The sums of the volumes of both testes were then divided by 2 to calculate the mean testicular volume.

### 2.3. Laboratory Assays

The biological material for laboratory assays was collected between 7.30 and 9.00 a.m. in a quiet and air-conditioned room. Urine samples were obtained using a sterile pediatric urine collection bag (Medicavera, Szczecin, Poland) attached by the parents over the child’s genitalia. Before collection, the genitals had been thoroughly cleaned and dried. At the end of the visit, the principal investigator (K.K.) collected saliva samples. A 2 mL sterile syringe was introduced gently into the mouth, and saliva was carefully aspirated from the floor of the mouth. To avoid the risk of contamination and to minimize the potential impact of feeding on sex hormone levels, the participants were not fed 1 h prior to the saliva collection. The procedure, lasting for 30–60 s, was not stressful for the infants. Empty and saliva-filled syringes were weighed, and the difference between weights was considered the amount of saliva collected. Urine and saliva samples were then frozen at −20 °C until analysis.

To minimize analytical errors, all assays were carried out in duplicate according to manufacturers’ instructions, and the final results were averaged. The measurements were performed by a person who was blinded to patient data and all clinical and diagnostic information. Urine creatinine was determined by Jaffe’s reaction using reagents obtained from Roche Diagnostics (Basel, Switzerland). FSH and LH levels in urine were measured by a solid-phase, two-site chemiluminescent immunometric assay (Siemens Healthcare Diagnostics, Erlangen, Germany), and the obtained results were corrected for creatinine to adjust for urine dilution. Salivary levels of testosterone, androstenedione, dehydroepiandrosterone sulfate (DHEA-S), estradiol, progesterone, and 17-hydroxyprogesterone were determined using an enzyme-linked immunosorbent assay (Diametra, Perugia, Italy; BioVendor R&D, Brno, Czech Republic; and IBL International, Hamburg, Germany). Detection limits are shown in Table 1.

### 2.4. Statistical Analysis

The LOD value was assigned for results below LOD if other results exceeded the limit. If all results were below LOD, no statistical analysis was performed. Prior to statistical analysis, all variables were tested for normality using Shapiro–Wilk’s test, and variables that were positively skewed were log-transformed. Within-group comparisons were made using a repeated-measures analysis of variance test, followed by Tukey’s post hoc test for pairwise comparisons. Inter-group comparisons at the same time point were performed using a one-way analysis of variance followed by Bonferroni’s test. Differences in the distribution of categorical variables were analyzed using Person’s chi-square test. The strength of relationships between the measured variables was estimated using Pearson’s r tests for two continuous variables, the phi coefficient for one continuous and one categorical variable, and point-biserial for two categorical variables. The result was considered statistically significant if the probability value (*p*) corrected for multiple comparisons was less than 0.05.

## 3. Results

**Figure 1:**
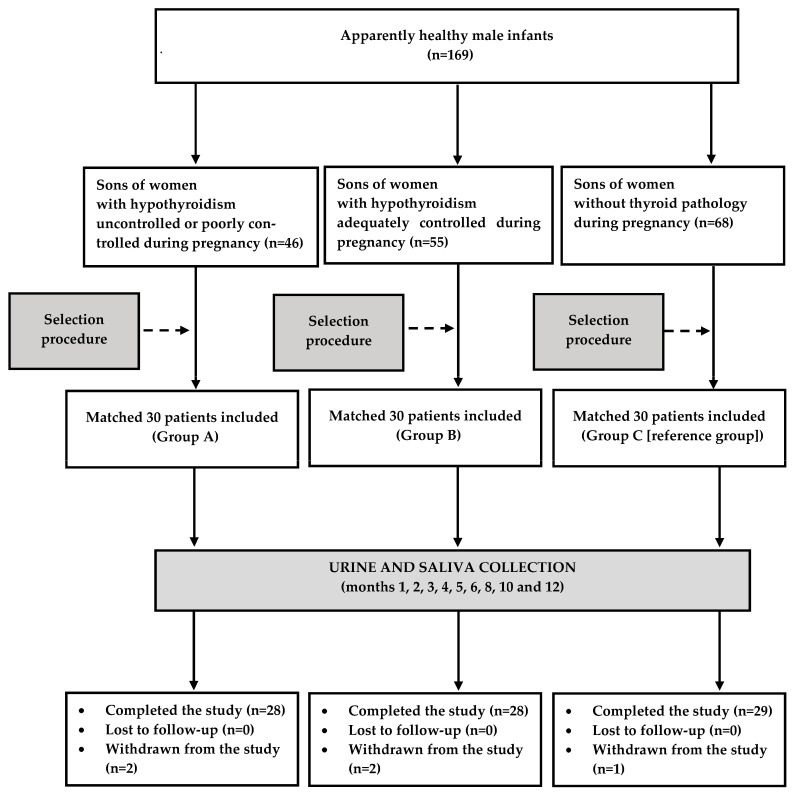
Flow the patients through the study.

A total of 169 boys met all inclusion and exclusion criteria. After performing the selection procedure, each study group consisted of 30 infants. Two patients assigned to group A were withdrawn from the study because of the development of vesicoureteral reflux and infantile atopic dermatitis. Owing to frequent acute infections, the required minimum number of samples was not obtained from two other boys (one from group B and one from group C). Lastly, one patient assigned to group B dropped out because of consent withdrawal. Eighty-five out of 90 participants (94%) completed the study, which means that the study was sufficiently powered. The flow of patients through the study is depicted in Figure 1.

There were no differences in age, education, occupational activity, type of work, the number of deliveries, body mass index, systolic blood pressure, and diastolic blood pressure between mothers of boys participating in the study in both intent-to-treat (Table 2) and per protocol (Supplementary Table S1) analyses. The mean maternal TSH concentration was higher in group A than in the remaining groups. The percentage of smoking mothers was similar in all three study groups. There were no differences in the reasons for hypothyroidism. Autoimmune thyroiditis was diagnosed in 15 mothers of patients from group A and 16 mothers of patients from group B, thyroidectomy in 10 mothers from group A and 9 mothers from group B, thyroid hypoplasia in 4 mothers from group A and 3 mothers from group B, while dyshormonogenesis in one mother from group A and 2 mothers from group B. In group A, similar numbers of boys were born to mothers with untreated (n = 14) or undertreated (n = 16) hypothyroidism. The number of untreated and undertreated mothers was the same (n = 14) in the case of children who completed the study.

There were no differences between the studied groups of infants in gestational age of delivery, birth order, length, weight, body mass index, head circumference, and TSH levels in both intent-to-treat (Table 3) and per protocol (Supplementary Table S2) analyses. The percentage of breast-fed infants was similar in all three groups.

In group A, testosterone was detectable in saliva for the first five months of life, while in the remaining groups, it was detectable for the first six months of life. In groups B and C, the highest concentrations of this hormone were observed at the end of the second month of life and decreased thereafter. In group A, testosterone remained at similar levels during the first four months of life. During the first six months of life, salivary testosterone levels were lower in group A than in groups B and C (Table 4).

FSH was detectable in urine samples for the first 8 months of life, during which there were lower in group A than in groups B and C, with no differences between the latter two groups. In all study groups, urinary FSH remained at similar levels until the age of month 5 and declined thereafter (Table 5).

Urinary LH levels were detectable for the first 6 months of life. Until the age of month 6, they were lowest in group A, while there were no differences in this parameter between groups B and C. Peak concentrations of this hormone were observed earlier (the end of the second month of life) in groups B and C than in group A (the end of the third month of life) (Table 6).

In group A, androstenedione was detectable in saliva for the first five months, while in groups B and C, it was detectable for the first six months of life. In each study group, stable levels of this hormone were observed for the first four months of life and decreased thereafter. Estradiol was detectable in saliva only during the first three months of life. DHEA-S, progesterone, and 17-hydroxyprogesterone were detectable in saliva throughout the study period. There were no between-group differences in salivary concentrations of androstenedione, estradiol, DHEA-S, progesterone, and 17-hydroxyprogesterone (Supplementary Tables S3–S7).

In all study groups, penile length increased with time. From postnatal month 5, penile length was greater in groups B and C than in group A. There were no differences in this parameter between groups B and C (Table 7).

Testicular volume increased to month 5 and, except for a decrease in group C after 12 months of life, remained stable thereafter. From postnatal month 5, testicular volume was greater in groups B and C than in group A. There were no differences in this parameter between groups B and C (Table 8).

Testosterone, FSH, and LH concentrations did not differ between women in whom hypothyroidism was uncontrolled or poorly controlled during pregnancy, resulting from autoimmune thyroiditis or thyroidectomy, thyroid hypoplasia, and dyshormonogenesis (Table 9).

During the first six months, there were positive correlations between salivary testosterone levels and urinary LH levels. These correlations were stronger in group B (r values between 0.49 [*p* = 0.0001] and 0.65 [*p* < 0.0001] depending on time point) and group C (r values between 0.51 [*p* < 0.0001] and 0.69 [*p* < 0.0001] depending on time point) than in group A (r values between 0.32 [*p* = 0.0204] and 0.46 [*p* = 0.0002] depending on time point). Moreover, testosterone levels stronger correlated with penile length in group B (r values between 0.35 [*p* = 0.0125] and 0.43 [*p* = 0.0008] depending on time point) and in group C (r values between 0.37 [*p* = 0.0183] and 0.44 [*p* = 0.0006] depending on time point) than in group A (r values between 0.28 [*p* = 0.0488] and 0.35 [*p* = 0.0148] depending on time point). In groups B and C, but not in group A, there were positive correlations between testicular volume and concentrations of FSH (group B: r values between 0.39 [*p* = 0.0015] and 0.47 [*p* = 0.0002] depending on time point; group C: r values between 0.40 [*p* = 0.0011] and 0.48 [*p* = 0.0001] depending on time point). In the subgroup of women with autoimmune thyroiditis, hormone levels did not correlate with thyroid antibody titers.

## 4. Discussion

Our study shows for the first time that maternal thyroid status during gestation impacts the course of minipuberty in the male offspring. Boys born to women with uncontrolled or poorly controlled hypothyroidism during gestation are characterized by lower levels of gonadotropins and testosterone and by less pronounced penile growth and testicular enlargement in the first months after the birth than sons of women with normal thyroid function.

Lower values of testosterone and LH and positive correlations between both hormones suggest that changes in their production in descendants of mothers with untreated or undertreated hypothyroidism reflect decreased activity of the hypothalamic-pituitary-testicular axis. The low threshold value of TSH above which hypothyroidism was considered inadequately controlled suggests that changes in postnatal hypothalamic-pituitary-testicular axis activation may be observed even in sons born to mothers with mild thyroid hypofunction during pregnancy. Thus, our findings may serve as an argument in favor of the routine assessment of TSH in all healthy pregnant women and the treatment of even mild forms of thyroid hypofunction. Because no study investigated the impact of other chronic disorders in pregnancy on hormonal changes during the first several months of life, it is difficult to conclude whether impaired activation of the reproductive axis is limited to the offspring of women with thyroid disorders or is a non-specific response to long-term changes in fetal homeostasis resulting from the presence of chronic maternal disorder.

Although observed in the whole study population, penile growth and testicular enlargement were more pronounced in descendants of women with unaltered thyroid function. Moreover, sons of women with uncontrolled or inadequately controlled hypothyroidism were characterized by weaker correlations between penile length and testosterone levels than the remaining groups of infant boys and by the lack of correlations between testicular volume and FSH, observed in sons born to healthy mothers and to mothers with compensated thyroid function. Some previous findings support the association between thyroid and testicular function. Sertoli cells express biologically active isoforms of the thyroid hormone receptor, and it has been found that thyroid hormones impact Sertoli cell proliferation in the early stages of postnatal life and possibly also during gestation [20]. Moreover, thyroid hormones were found to modulate testosterone production and activity of the androgen receptor [21]. Maternal thyroid hormones penetrate the placental barrier and represent an important pool of thyroid hormones in the fetal circulation, particularly in the first half of pregnancy [17]. Thus, low levels of these hormones at the early stages of gestation (when fetal thyroid secretory function is low) may have an unfavorable effect on the first phase of the gonadal axis activation. This phase, taking place in utero between the 10th and 24th gestational week, is postulated to play a role in normal genital development [22,23].

Interestingly, there were no differences in the investigated hormones (as well as in penile length and testicular volume) between sons of healthy women and sons of women in whom normal thyroid function was a consequence of levothyroxine substitution. This finding indicates that the course of male minipuberty is undisturbed if the mother is effectively treated during pregnancy. The obtained results support the rationale of threshold values for pharmacological interventions suggested by the American Thyroid Association [17]. In turn, similar etiology of thyroid hypofunction in untreated/undertreated and adequately treated mothers suggests that the observed changes in hypothalamic-pituitary-testicular axis activity result from low thyroid status during gestation and do not seem to be specific to the underlying disorder.

We did not observe differences in gonadotropin and testosterone levels between sons of women with autoimmune and non-autoimmune hypothyroidism. Moreover, salivary or urinary levels of the investigated hormones did not correlate with maternal titers of thyroid peroxidase and thyroglobulin antibodies, elevated values of which are characteristic for individuals with Hashimoto thyroiditis and correlate with the severity of autoimmune thyroid destruction [24]. Thus, the obtained results argue against the association between changes in the hormonal profile of male infants and thyroid autoimmunity. Because thyroid peroxidase and thyroglobulin antibodies belong to the immunoglobulin G class of antibodies, they pass the placental barrier and are often detectable in fetal plasma during pregnancy and during the first several weeks of postnatal life [24]. However, they do not seem to have a meaningful impact on fetal and neonatal thyroid function [25], and our findings are in line with this commonly accepted view.

Our study has also shown that estradiol was stably detectable in saliva during the first three months of life. A shorter period of detection and much lower salivary levels in comparison with testosterone and the remaining androgens probably reflected differences in sex hormone production, resulting from low aromatase activity in males before puberty [26]. However, no between-group differences in estradiol levels and the lack of correlations with the remaining outcome variables argue against the role of estradiol in the development of male genital organs in the first months of life and suggest that estrogen production and metabolism in infant boys are unaffected by the mother’s thyroid status during gestation.

The obtained results allow us to draw other conclusions. First, the measurement of sex hormones in saliva may be an alternative for their assessment in plasma or serum because saliva collection is pain-free and much less stressful in comparison with blood collection. Moreover, owing to abundant production in infancy, it is easy to collect enough saliva, allowing the simultaneous assessment of several steroid hormones. Second, because of fluctuations, the reference values for salivary testosterone, androstenedione, and estradiol in boys in the first year of life should be age-specific and assay-method-specific. Lastly, the presence of detectable concentrations of DHEA-S, progesterone, and 17-hydroxyprogesterone over the entire study period suggests that their assessment in saliva may be useful in the diagnosis and control of congenital adrenal hypoplasia, one of the most common inherited metabolic disorders, which is characterized by increased production of all these steroids [27].

Among boys considered for enrollment, 27% had mothers with hypothyroidism untreated or undertreated during pregnancy. The high percentage of such females seems to result from an inaccurate approach of some physicians to thyroid hypofunction during gestation in real-life, everyday clinical practice. This observation is also in line with the recent data from other research centers. A population-based cohort study conducted in the United Kingdom showed that between 1998 and 2017, only 10% of women with subclinical hypothyroidism were prescribed levothyroxine during pregnancy [28]. In the United States, only 52.6% of pregnant women with hypothyroidism were treated in a manner consistent with American Thyroid Association guidelines, while the remaining ones were untreated (23.9%), overtreated (1.0%) or did not have TSH monitored during pregnancy (22.5%) [29]. Lastly, only 50.3% of pregnant women diagnosed with hypothyroidism in Spain received thyroid hormone substitution, and only 54% of levothyroxine-substituted women presented high adherence during pregnancy [30]. All these findings clearly indicate the need for improvement in the diagnosis and treatment of this disorder during gestation and women desiring pregnancy.

Some study limitations need to be highlighted. The study included only boys with negative screening test results for congenital hypothyroidism and without sonographic features of thyroid dysgenesis. Because we did not collect peripheral blood samples to assess TSH and thyroid hormone levels, we cannot fully exclude mild or transient disturbances in thyroid function in the participants. Although the study had sufficient power to detect presumed differences in the primary endpoint, the small number of participants decreased the reliability of the results. Our research was a cohort study, and therefore, its findings might have been influenced by the impact of latent confounders and selection bias. In our study, the adequacy and inadequacy of levothyroxine substitution were defined based on arbitrary criteria, which might have affected the obtained results. Although immunoassays used in the current study are widely used in daily practice, mass spectrometry-based methods are more specific and regarded as the gold standard in the measurement of steroids [31]. The study was carried out in the area of adequate iodine [32] and inadequate selenium [33] intake. It is not certain whether the impact of maternal hypothyroidism on the course of minipuberty is the same in patients with low iodine and/or adequate selenium status. Lastly, although the study design minimized the impact of random diurnal, seasonal, and analytical variations in the investigated hormones, the obtained results might have been influenced by the regression-to-the-mean effect [34].

## 5. Conclusions

The results of our study indicate that untreated or undertreated maternal hypothyroidism during pregnancy may have an unfavorable effect on postnatal activation of the reproductive axis and the development of male genital organs. They suggest that thyroid hypofunction, even mild and asymptomatic/oligosymptomatic, should be effectively controlled throughout pregnancy. The novelty of the obtained results, as well as the limitations of the study design and methodology cause them to need to be verified in future larger-scale, multicenter, controlled studies. Our findings should also be an incentive to investigate the course of minipuberty in daughters born to hypothyroid women and in the offspring of mothers with other chronic disorders.

## Figures and Tables

**Table 1:** Limits of detection of assessed variables.

Hormone	Material	Value
Testosterone	Saliva	10 pmol/L
Androstenedione	Saliva	18 pmol/L
DHEA-S	Saliva	100 nmol/L
Estradiol	Saliva	4 pmol/L
Progesterone	Saliva	16 pmol/L
17-hydroxyprogesterone	Saliva	11 pmol/L
FSH	Urine	0.1 U/L
LH	Urine	0.1 U/L

Abbreviations: DHEA-S—dehydroepiandrosterone sulfate; FSH—follicle-stimulating hormone; LH—luteinizing hormone.

**Table 2:** Characteristics of mothers of patients participating in the study.

Variable	Group A	Group B	Group C	*p*-Value		
				A vs. B	A vs. C	B vs. C
Number (n)	30	30	30	-	-	-
Age (years)	31 ± 7	32 ± 7	31 ± 8	0.5822	1.0000	0.6083
Primary or vocational/secondary/university education (%)	27/37/37	23/37/37	23/37/40	0.8235	0.7554	0.7448
Occupational activity/white-collar/pink-collar/blue-collar workers (%)	77/23/30/23	80/23/27/30	77/27/27/23	0.7568	0.8115	0.8205
Number of deliveries (n)	1.5 ± 0.6	1.6 ± 0.5	1.6 ± 0.6	0.4859	0.5212	1.0000
Smokers (%)	23	20	23	-	-	-
Body mass index (kg/m^2^)	24.8 ± 3.7	24.1 ± 3.5	24.3 ± 3.9	0.4546	0.6124	0.8351
Systolic blood pressure (mmHg)	124 ± 17	121 ± 15	119 ± 19	0.4715	0.2872	0.6525
Diastolic blood pressure (mmHg)	81 ± 6	79 ± 6	79 ± 5	0.2018	0.1661	1.000
TSH (mU/L)	7.5 ± 2.8	1.5 ± 0.8	1.6 ± 0.8	<0.0001	<0.0001	0.6301

Unless otherwise stated, the data are presented as the mean ± standard deviation. Body mass index, blood pressure, and TSH levels represent mean values from visits during which TSH concentrations were registered. Group A: sons of women with hypothyroidism uncontrolled or poorly controlled during pregnancy; Group B: sons of women with hypothyroidism adequately controlled during pregnancy; Group C: sons of healthy women.

**Table 3:** Baseline characteristics of male newborns participating in the study.

Variable	Group A	Group B	Group C	*p*-Value		
				A vs. B	A vs. C	B vs. C
Number (n)	30	30	30	-	-	-
Gestational age of delivery (weeks)	39 ± 2	40 ± 2	40 ± 2	0.1052	0.1052	1.0000
Birth order: first/second/third and subsequent (%)	50/40/10	47/47/6	47/43/10	0.5286	0.6148	0.6885
Length (cm)	54.3 ± 1.7	54.7 ± 1.6	54.4 ± 1.5	0.3519	0.8095	0.4568
Weight (kg)	4.51 ± 0.49	4.53 ± 0.53	4.60 ± 0.51	0.8798	0.4886	0.6042
Body mass index (kg/m^2^)	15.2 ± 0.8	15.1 ± 1.1	15.5 ± 0.8	0.6887	0.1518	0.1342
Head circumference (cm)	37.4 ± 0.6	37.2 ± 0.7	37.1 ± 0.9	0.2396	0.1342	0.6324
Breast-feeding (%)	80	83	87	-	-	-
TSH (mU/L)	7.1 ± 2.8	6.8 ± 2.3	6.9 ± 2.4	0.6519	0.7675	0.8697

Unless otherwise stated, the data are presented as the mean ± standard deviation. TSH was measured in dried whole blood spots obtained on days 3–5 of life. Group A: sons of women with hypothyroidism uncontrolled or poorly controlled during pregnancy; Group B: sons of women with hypothyroidism adequately controlled during pregnancy; Group C: sons of healthy women.

**Table 4:** Salivary testosterone levels in the study population.

Age	Group A	Group B	Group C	*p*-Value		
				A vs. B	A vs. C	B vs. C
1 month	104 ± 32	160 ± 48	172 ± 50	<0.0001	<0.0001	0.3637
2 months	112 ± 45	204 ± 53 ^a^	212 ± 62 ^a^	<0.0001	<0.0001	0.6059
3 months	120 ± 55	172 ± 43 ^b^	178 ± 55 ^b^	0.0002	0.0002	0.6489
4 months	125 ± 58	155 ± 40 ^b^	160 ± 62 ^b^	0.0283	0.0321	0.7200
5 months	55 ± 20 ^a,b,c,d^	120 ± 60 ^a,b,c,d^	115 ± 56 ^a,b,c,d^	<0.0001	<0.0001	0.7461
6 months	Below LOD ^a,b,c,d,e^	40 ± 24 ^a,b,c,d,e^	46 ± 20 ^a,b,c,d,e^	<0.0001	<0.0001	0.3090
8 months	Below LOD	Below LOD	Below LOD	-	-	-
10 months	Below LOD	Below LOD	Below LOD	-	-	-
12 months	Below LOD	Below LOD	Below LOD	-	-	-

The data are expressed in pmol/L and presented as the mean ± standard deviation. Only samples of patients who completed the study were analyzed. To perform statistical analyses, the LOD value (10 pmol/L) was assigned for testosterone in group A at the age of 6 months. Group A: sons of women with hypothyroidism uncontrolled or poorly controlled during pregnancy; Group B: sons of women with hypothyroidism adequately controlled during pregnancy; Group C: sons of healthy women. ^a^ *p* < 0.05 vs. levels at the age of 1 month in the same study group; ^b^ *p* < 0.05 vs. levels at the age of 2 months in the same study group; ^c^ *p* < 0.05 vs. levels at the age of 3 months in the same study group; ^d^ *p* < 0.05 vs. levels at the age of 4 months in the same study group; ^e^ *p* < 0.05 vs. levels at the age of 5 months in the same study group. Abbreviation: LOD—limit of detection.

**Table 5:** Urinary FSH levels in the study population.

Age	Group A	Group B	Group C	*p*-Value		
				A vs. B	A vs. C	B vs. C
1 month	0.71 ± 0.24	0.92 ± 0.23	0.88 ± 0.25	0.0015	0.0114	0.5326
2 months	0.68 ± 0.22	0.98 ± 0.38	0.95 ± 0.30	0.0006	0.0003	0.7416
3 months	0.73 ± 0.19	0.90 ± 0.32	0.97 ± 0.34	0.0190	0.0018	0.4273
4 months	0.65 ± 0.20	0.86 ± 0.35	0.84 ± 0.28	0.0194	0.0165	0.8123
5 months	0.70 ± 0.28	0.92 ± 0.32	0.89 ± 0.31	0.0084	0.0186	0.7206
6 months	0.48 ± 0.18 ^a,b,c,d,e^	0.69 ± 0.25 ^a,b,c,d,e^	0.64 ± 0.28 ^a,b,c,d,e^	0.0007	0.0134	0.4805
8 months	0.29 ± 0.20 ^a,b,c,d,e,f^	0.46 ± 0.24 ^a,b,c,d,e,f^	0.42 ± 0.23 ^a,b,c,d,e,f^	0.0069	0.0269	0.5232
10 months	Below LOD	Below LOD	Below LOD	-	-	-
12 months	Below LOD	Below LOD	Below LOD	-	-	-

The data are expressed in international units per µmol of creatinine and presented as the mean ± standard deviation. Only samples of patients who completed the study were analyzed. Group A: sons of women with hypothyroidism uncontrolled or poorly controlled during pregnancy; Group B: sons of women with hypothyroidism adequately controlled during pregnancy; Group C: sons of healthy women. ^a^ *p* < 0.05 vs. levels at the age of 1 month in the same study group; ^b^ *p* < 0.05 vs. levels at the age of 2 months in the same study group; ^c^ *p* < 0.05 vs. levels at the age of 3 months in the same study group; ^d^ *p* < 0.05 vs. levels at the age of 4 months in the same study group; ^e^ *p* < 0.05 vs. levels at the age of 5 months in the same study group; ^f^ *p* < 0.05 vs. levels at the age of 6 months in the same study group. Abbreviations: FSH—follicle-stimulating hormone; LOD—limit of detection.

**Table 6:** Urinary LH levels in the study population.

Age	Group A	Group B	Group C	*p*-Value		
				A vs. B	A vs. C	B vs. C
1 month	0.97 ± 0.38	1.65 ± 0.55	1.70 ± 0.60	<0.0001	<0.0001	0.7440
2 months	1.02 ± 0.40	1.95 ± 0.50 ^a^	2.04 ± 0.56 ^a^	<0.0001	<0.0001	0.5722
3 months	1.24 ± 0.46 ^a,b^	1.64 ± 0.58 ^b^	1.67 ± 0.64 ^b^	0.0060	0.0053	0.8537
4 months	0.82 ± 0.42 ^c^	1.34 ± 0.50 ^a,b,c^	1.30 ± 0.58 ^a,b,c^	0.0001	0.0008	0.7817
5 months	0.60 ± 0.38 ^a,b,c,d^	1.10 ± 0.42 ^a,b,c,d^	1.03 ± 0.46 ^a,b,c,d^	<0.0001	0.0003	0.5514
6 months	0.38 ± 0.20 ^a,b,c,d,e^	0.65 ± 0.30 ^a,b,c,d,e^	0.72 ± 0.26 ^a,b,c,d,e^	0.0002	<0.0001	0.3501
8 months	Below LOD	Below LOD	Below LOD	-	-	-
10 months	Below LOD	Below LOD	Below LOD	-	-	-
12 months	Below LOD	Below LOD	Below LOD	-	-	-

The data are expressed in international units per µmol of creatinine and presented as the mean ± standard deviation. Only samples of patients who completed the study were analyzed. Group A: sons of women with hypothyroidism uncontrolled or poorly controlled during pregnancy; Group B: sons of women with hypothyroidism adequately controlled during pregnancy; Group C: sons of healthy women. ^a^ *p* < 0.05 vs. levels at the age of 1 month in the same study group; ^b^ *p* < 0.05 vs. levels at the age of 2 months in the same study group; ^c^ *p* < 0.05 vs. levels at the age of 3 months in the same study group; ^d^ *p* < 0.05 vs. levels at the age of 4 months in the same study group; ^e^ *p* < 0.05 vs. levels at the age of 5 months in the same study group. Abbreviations: LH—luteinizing hormone; LOD—limit of detection.

**Table 7:** Penile length in the participants of the study.

Age	Group A	Group B	Group C	*p*-Value		
				A vs. B	A vs. C	B vs. C
1 month	3.6 ± 0.4	3.5 ± 0.5	3.6 ± 0.4	0.4122	1.0000	0.4072
2 months	3.6 ± 0.4	3.6 ± 0.3	3.7 ± 0.5	1.0000	0.3660	0.4090
3 months	3.7 ± 0.5	3.7 ± 0.4	3.8 ± 0.4	1.0000	0.4072	0.3495
4 months	3.7 ± 0.5	3.8 ± 0.3 ^a,b^	3.9 ± 0.4 ^a,b^	0.3682	0.1108	0.2916
5 months	3.7 ± 0.4	4.0 ± 0.3 ^a,b,c^	4.0 ± 0.5 ^a,b^	0.0025	0.0156	1.0000
6 months	3.7 ± 0.4	4.1 ± 0.4 ^a,b,c,d^	4.2 ± 0.4 ^a,b,c,d^	0.0004	<0.0001	0.3495
8 months	3.8 ± 0.5	4.2 ± 0.5 ^a,b,c,d^	4.3. ± 0.4 ^a,b,c,d^	0.0042	0.0001	0.4072
10 months	3.8 ± 0.4 ^a,b^	4.2 ± 0.4 ^a,b,c,d^	4.3 ± 0.4 ^a,b,c,d^	0.0004	<0.0001	0.3515
12 months	3.9 ± 0.4 ^a,b^	4.3 ± 0.4 ^a,b,c,d,e,f^	4.4 ± 0.5 ^a,b,c,d,e^	0.0005	0.0001	0.4090

The data are expressed in cm and presented as the mean ± standard deviation. Only samples of patients who completed the study were analyzed. Group A: sons of women with hypothyroidism uncontrolled or poorly controlled during pregnancy; Group B: sons of women with hypothyroidism adequately controlled during pregnancy; Group C: sons of healthy women. ^a^ *p* < 0.05 vs. values at the age of 1 month in the same study group; ^b^ *p* < 0.05 vs. values at the age of 2 months in the same study group; ^c^ *p* < 0.05 vs. values at the age of 3 months in the same study group; ^d^ *p* < 0.05 vs. values at the age of 4 months in the same study group; ^e^ *p* < 0.05 vs. values at the age of 5 months in the same study group; ^f^ *p* < 0.05 vs. values at the age of 6 months in the same study group.

**Table 8:** Testicular volume in the participants of the study.

Age	Group A	Group B	Group C	*p*-Value		
				A vs. B	A vs. C	B vs. C
1 month	0.30 ± 0.07	0.31 ± 0.06	0.30 ± 0.07	0.5684	1.0000	0.5655
2 months	0.32 ± 0.05	0.34 ± 0.07	0.32 ± 0.06	0.2239	1.0000	0.2513
3 months	0.32 ± 0.08	0.35 ± 0.05 ^a^	0.34 ± 0.04 ^a^	0.0920	0.2352	0.4072
4 months	0.34 ± 0.09	0.38 ± 0.05 ^a,b^	0.37 ± 0.06 ^a,b^	0.0614	0.4978	0.3706
5 months	0.35 ± 0.06 ^a^	0.42 ± 0.05 ^a,b,c,d^	0.43 ± 0.05 ^a,b,c,d^	<0.0001	<0.0001	0.4535
6 months	0.38 ± 0.04 ^a,b,c^	0.44 ± 0.06 ^a,b,c,d^	0.43 ± 0.10 ^a,b,c,d^	0.0001	0.0170	0.6504
8 months	0.37 ± 0.06^.a,b,c^	0.43 ± 0.09 ^a,b,c,d^	0.42 ± 0.06 ^a,b,c,d^	0.0049	0.0027	0.6224
10 months	0.38 ± 0.07 ^a,b,c^	0.43 ± 0.06 ^a,b,c,d^	0.42 ± 0.05 ^a,b,c,d^	0.0053	0.0159	0.4965
12 months	0.36 ± 0.06 ^a,b,c^	0.41 ± 0.07 ^a,b,c,d^	0.39 ± 0.06 ^a,b,c,d,e^	0.0059	0.2513	0.0495

The data are expressed in mL and presented as the mean ± standard deviation. Only samples of patients who completed the study were analyzed. Group A: sons of women with hypothyroidism uncontrolled or poorly controlled during pregnancy; Group B: sons of women with hypothyroidism adequately controlled during pregnancy; Group C: sons of healthy women. ^a^ *p* < 0.05 vs. values at the age of 1 month in the same study group; ^b^ *p* < 0.05 vs. values at the age of 2 months in the same study group; ^c^ *p* < 0.05 vs. values at the age of 3 months in the same study group; ^d^ *p* < 0.05 vs. values at the age of 4 months in the same study group; ^e^ *p* < 0.05 vs. values at the age of 5 months in the same study group.

**Table 9:** Testosterone and gonadotropin levels in sons of women with hypothyroidism uncontrolled or poorly controlled during pregnancy of autoimmune or non-autoimmune origin.

Age	Autoimmune Hypothyroidism	Non-Autoimmune Hypothyroidism	*p*-Value
	Testosterone		
1 month	88 ± 34	112 ± 43	0.0654
2 months	96 ± 42	103 ± 27	0.4614
3 months	105 ± 29	95 ± 23	0.1586
4 months	108 ± 35	92 ± 24^,^	0.0622
5 months	102 ± 32	98 ± 19	0.5715
	FSH		
1 month	92 ± 39	108 ± 40	0.1455
2 months	98 ± 28	101 ± 34	0.7198
3 months	103 ± 42	97 ± 25	0.5187
4 months	110 ± 43	90 ± 35	0.0715
5 months	101 ± 32	98 ± 30	0.7188
6 months	95 ± 26	106 ± 37	0.2035
8 months	101 ± 30	100 ± 24	0.8910
	LH		
1 month	91 ± 35	109 ± 46	0.1013
2 months	104 ± 38	96 ± 30	0.3774
3 months	98 ± 22	102 ± 25	0.5206
4 months	95 ± 21	105 ± 30	0.1470
5 months	97 ± 28	103 ± 25	0.3940
6 months	104 ± 31	96 ± 28	0.3068

The data are expressed as the percentage of the mean value in the whole group A ± standard deviation. Only samples of patients who completed the study were analyzed. Abbreviations: FSH—follicle-stimulating hormone; LH—luteinizing hormone.

## Data Availability

The data that support the findings of this study are available from the corresponding author upon reasonable request.

## Supplementary Materials

The following supporting information can be downloaded at: https://www.mdpi.com/article/10.3390/jcm12247649/s1, Table S1: Characteristics of mothers of patients who completed the study; Table S2: Baseline characteristics of male infants who completed the study; Table S3: Salivary androstenedione levels in the study population; Table S4: Salivary estradiol levels in the study population; Table S5: Salivary DHEA-S levels in the study population; Table S6: Salivary progesterone levels in the study population; Table S7: Salivary 17-hydroxyprogesterone levels in the study population.

## Abbreviations

DHEA-S	dehydroepiandrosterone sulfate
FSH	follicle-stimulating hormone
LH	luteinizing hormone
LOD	limit of detection
TSH	thyroid-stimulating hormone
