# Supplementary material for: The Impact of Maternal Hypothyroidism during Pregnancy on Minipuberty in Boys
Source: J Clin Med. 2023 Dec 13;12(24):7649. doi: 10.3390/jcm12247649 (PMC10744195; doi:10.3390/jcm12247649)
Supplement: Supplementary file 1 [file jcm-12-07649-s001.zip › jcm-2667756-supplementary.pdf]

Supplementary Table S1. Characteristics of mothers of patients who completed the study

Variable	Group A	Group B	Group C	*p*-value		
				A *vs.* B	A *vs.* C	B *vs.* C
Number (n)	28	28	29	-	-	-
Age (years)	32±7	31±8	30±8	0.6207	0.3202	0.6909
Primary or vocational/secondary/university education (%)	25/39/36	25/36/39	21/38/41	0.8524	0.7324	0.7640
Occupational activity/white-collar/pink-collar/blue-collar workers (%)	75/21/29/25	79/21/29/29	76/24/28/24	0.7875	0.8312	0.8422
Number of deliveries (n)	1.6±0.7	1.6±0.6	1.7±0.7	1.0000	0.5913	0.5655
Smokers (%)	21	21	24	-	-	-
Body mass index (kg/m^2)	24.6±3.5	24.0±3.8	24.2±4.0	0.5414	0.6898	0.8474
Systolic blood pressure (mmHg)	123±18	120±16	118±20	0.5126	0.3261	0.6790
Diastolic blood pressure (mmHg)	81±7	80±6	79±6	0.5684	0.2513	0.5319
TSH (mU/L)	7.6±2.9	1.6±0.8	1.7±0.8	<0.0001	<0.0001	0.6389

Unless otherwise stated, the data are presented as the mean ± standard deviation. Body mass index and blood pressure represent mean values from visits during which TSH concentrations were registered. Group A: sons of women with hypothyroidism uncontrolled or poorly controlled during pregnancy; Group B: sons of women with hypothyroidism adequately controlled during pregnancy; Group C: sons of healthy women.

Supplementary Table S2. Baseline characteristics of male infants who completed the study

Variable	Group A	Group B	Group C	p-value		
				A *vs.* B	A *vs.* C	B *vs.* C
Number (n)	28	28	29	-	-	-
Gestational age of delivery (weeks)	39±2	39±2	40±2	1.0000	0.1568	0.1568
Birth order: first/second/third and subsequent (%)	46/43/11	43/50/7	42/48/10	0.5056	0.5255	0.4876
Length (cm)	54.1±1.5	54.4±1.7	54.3±1.6	0.6286	0.4835	0.8188
Weight (kg)	4.48±0.51	4.56±0.52	4.61±0.49	0.5635	0.7100	0.3286
Body mass index (kg/m^2)	15.3±0.9	15.4±1.0	15.6±0.8	0.6956	0.4072	0.2064
Head circumference (cm)	37.2±0.7	37.1±0.6	37.0±0.6	0.9544	0.9081	0.9501
Breastfeeding (%)	79	86	83	-	-	-
TSH (mU/L)	7.2±2.6	6.9±2.4	6.8±2.2	0.6555	0.5327	0.8703

Unless otherwise stated, the data are presented as the mean ± standard deviation. TSH was measured in dried whole blood spots, obtained on day 3-5 of life. Group A: sons of women with hypothyroidism uncontrolled or poorly controlled during pregnancy; Group B: sons of women with hypothyroidism adequately controlled during pregnancy; Group C: sons of healthy women.

Supplementary Table S3. Salivary androstenedione levels in the study population

Age	Group A	Group B	Group C
1 month	82±35	93±42	98±43
2 months	90±40	105±40	96±46
3 months	95±46	111±50	108±44
4 months	100±48	98±41	90±46
5 months	51±29[a,b,c,d]	62±35 [a,b,c,d]	55±20 [a,b,c,d]
6 months	Below LOD [a,b,c,d,e]	30±22 [a,b,c,d,e]	28±25 [a,b,c,d,e]
8 months	Below LOD	Below LOD	Below LOD
10 months	Below LOD	Below LOD	Below LOD
12 months	Below LOD	Below LOD	Below LOD

The data are expressed in pmol/L and presented as the mean ± standard deviation. Only samples of patients who completed the study were analyzed. To performed statistical analyses, LOD value (18 pmol/L) was assigned for androstenedione in group A at the age of 6 months. Group A: sons of women with hypothyroidism uncontrolled or poorly controlled during pregnancy; Group B: sons of women with hypothyroidism adequately controlled during pregnancy; Group C: sons of healthy women. [a]$p<0.05$ *vs*. levels at the age of 1 month in the same study group; [b]$p<0.05$ *vs*. levels at the age of 2 months in the same study group; [c]$p<0.05$ *vs*. levels at the age of 3 months in the same study group; [d]$p<0.05$ *vs*. levels at the age of 4 months in the same study group; [e]$p<0.05$ *vs*. levels at the age of 5 months in the same study group. Abbreviation: LOD - limit of detection.

Supplementary Table S4. Salivary estradiol levels in the study population

Age	Group A	Group B	Group C
1 month	14±6	16±8	17±9
2 months	10±5	13±7	14±9
3 months	12±7	10±6	10±5
4 months	Below LOD	Below LOD	Below LOD
5 months	Below LOD	Below LOD	Below LOD
6 months	Below LOD	Below LOD	Below LOD
8 months	Below LOD	Below LOD	Below LOD
10 months	Below LOD	Below LOD	Below LOD
12 months	Below LOD	Below LOD	Below LOD

The data are expressed in pmol/L and presented as the mean ± standard deviation. Only samples of patients who completed the study were analyzed. Group A: sons of women with hypothyroidism uncontrolled or poorly controlled during pregnancy; Group B: sons of women with hypothyroidism adequately controlled during pregnancy; Group C: sons of healthy women. Abbreviation: LOD – limit of detection.

Supplementary Table S5. Salivary DHEA-S levels in the study population

Age	Group A	Group B	Group C
1 month	160±40	148±63	155±58
2 months	145±80	140±65	144±53
3 months	150±56	132±50	137±56
4 months	132±48	136±47	143±49
5 months	168±69	142±48	150±52
6 months	138±67	150±68	158±50
8 months	148±46	139±70	143±51
10 months	141±50	155±68	148±52
12 months	162±49	146±57	160±72

The data are expressed in nmol/L and presented as the mean ± standard deviation. Only samples of patients who completed the study were analyzed. Group A: sons of women with hypothyroidism uncontrolled or poorly controlled during pregnancy; Group B: sons of women with hypothyroidism adequately controlled during pregnancy; Group C: sons of healthy women. Abbreviations: DHEA-S - dehydroepiandrosterone sulfate

Supplementary Table S6. Salivary progesterone levels in the study population

Age	Group A	Group B	Group C
1 month	135±60	142±71	145±78
2 months	120±72	132±62	155±85
3 months	142±58	126±61	143±64
4 months	151±78	146±46	135±55
5 months	156±82	150±55	129±60
6 months	146±75	152±64	141±53
8 months	150±69	142±68	136±69
10 months	128±64	152±74	145±56
12 months	136±53	139±60	150±75

The data are expressed in pmol/L and presented as the mean ± standard deviation. Only samples of patients who completed the study were analyzed. Group A: sons of women with hypothyroidism uncontrolled or poorly controlled during pregnancy; Group B: sons of women with hypothyroidism adequately controlled during pregnancy; Group C: sons of healthy women.

Supplementary Table S7. Salivary 17-hydroxyprogesterone levels in the study population

Age	Group A	Group B	Group C
1 month	92±41	100±46	88±50
2 months	94±39	94±38	110±64
3 months	88±39	89±40	103±55
4 months	100±34	108±50	101±48
5 months	105±43	103±42	90±42
6 months	111±59	95±40	94±51
8 months	102±49	87±42	100±56
10 months	98±35	94±39	107±61
12 months	102±40	98±44	94±46

The data are expressed in pmol/L and presented as the mean ± standard deviation. Only samples of patients who completed the study were analyzed. Group A: sons of women with hypothyroidism uncontrolled or poorly controlled during pregnancy; Group B: sons of women with hypothyroidism adequately controlled during pregnancy; Group C: sons of healthy women.
